# Genetic variant in CXCL13 gene is associated with susceptibility to intrauterine infection of hepatitis B virus

**DOI:** 10.1038/srep26465

**Published:** 2016-05-23

**Authors:** Zhihua Wan, Xiaofang Lin, Tongyang Li, Aifen Zhou, Mei Yang, Dan Hu, Li Feng, Songxu Peng, Linlin Fan, Si Tu, Bin Zhang, Yukai Du

**Affiliations:** 1 Department of Maternal and Child Health and MOE (Ministry of Education) Key Laboratory of Environment and Health, School of Public Health, Tongji Medical College, Huazhong University of Science and Technology, Wuhan, Hubei, China; 2 Wuhan Women and Children Medical and Healthcare Center, Wuhan, Hubei, China

## Abstract

Intrauterine infection of hepatitis B virus (HBV), which accounts for the majority of mother-to-child transmission, is one of the main reasons for the failure of combined immunoprophylaxis against the transmission. Recent studies have identified that genetic background might influence the susceptibility to intrauterine infection of HBV. We conducted this study to investigate the associations between 10 genetic variants in 9 genes (SLC10A1, HLA-DP, HLA-C, CXCR5, CXCL13, TLR3, TLR4, TLR9 and UBE2L3) of mothers and their neonates and HBV intrauterine infection. A significantly decreased risk of HBV intrauterine transmission were found among mothers who carried the rs355687 CT genotypes in CXCL13 gene compared to those with CC genotypes (OR = 0.25, 95% CI, 0.08–0.82, *P* = 0.022); and a marginally significantly decreased risk was also observed under the dominant model (OR = 0.34, 95% CI, 0.11–1.01, *P* = 0.052). Besides, neonatal rs3130542 in HLA-C gene was found to be marginally significantly associated with decreased risk of HBV intrauterine infection under the additive model (OR = 0.55, 95% CI, 0.29–1.04, *P* = 0.064). However, we found no evidence of associations between the remaining 8 SNPs and risk of HBV intrauterine infection among mothers and their neonates. In conclusion, this study suggested that genetic variant in CXCL13 gene was associated with susceptibility to intrauterine infection of HBV.

Hepatitis B virus (HBV) infection is a global public health problem with 350 million people being chronically infected worldwide. People with hepatitis B are at increased risk of developing hepatic decompensation, cirrhosis, and hepatocellular carcinoma^1^. China is an endemic region, with a 7.2% prevalence rate of chronic HBV infection in individuals aged 1–59^2^. Mother-to-child transmission (MTCT), including intrauterine, labor, postnatal, is considered as one of the main modes of HBV infection. The rate of intrauterine transmission, which accounts for the majority of mother-to-child transmission, among HBsAg positive pregnant women ranges from 5% to 40% in different areas of China^3^,^4^. Infants with intrauterine HBV infection have a considerably high risk of becoming chronic carriers. Currently, combined immunoprophylaxis with the utilization of hepatitis B vaccine and hepatitis B immunoglobulin (HBIG) can block most cases of labor and postnatal transmission of HBV. Whereas, HBV intrauterine infection still occurs at high incidence due to lack of effective preventive measures, and its exact underlying mechanisms have not been completely elucidated. Therefore, exploring the risk factors of susceptibility to intrauterine HBV infection will help effectively control the spread of HBV infection among neonates.

Previous studies regarding the factors associated with HBV intrauterine infection were mostly focused on maternal characteristics (i.e. mode of delivery, maternal HBeAg status and viral load)^5^,^6^. In addition to maternal characteristics, genetic background may also play an important role in HBV intrauterine infection. However, few studies^7^,^8^,^9^ had explored the genetic susceptibility to intrauterine infection of HBV, and still, little is known about the associations between genetic factors and HBV intrauterine infection.

Sodium taurocholate cotransporting polypeptide (NTCP), identified as a functional receptor for human hepatitis B virus^10^, is encoded by the SLC10A1 gene. Variation of the p.S267F (rs2296651) in SLC10A1 gene results in loss of HBV receptor function *in vitro* and was found to be significantly associated with resistance to chronic hepatitis B^11^. Meanwhile, Zhenzhen Su *et al*.^12^ demonstrated that AA genotype of the other locus (rs7154439) in SLC10A1 gene was also associated with HBV clearance. Recently, genome-wide association studies (GWAS) in Japanese and Korean populations determined that single nucleotide polymorphisms (SNPs) in the HLA-DP gene were associated with persistent HBV infection^13^. Subsequently, one locus (rs3128917) in the HLA-DP was observed to be associated with chronic HBV infection in the Chinese population^14^. The other GWAS conducted in Han Chinese population had identified rs3130542 in HLA-C and rs4821116 in UBE2L3 to be associated with chronic HBV infection^15^. Follicular T helper (TfH) cells are newly recognized lineage of CD4^+^ T cells^16^, which have been reported to be partially responsible for the dysfunction of immune responses during HBV infection^17^. CXCR5 and CXCL13 are two of TfH-associated genes, and genetic variants in the CXCR5 gene (rs3922) and CXCL13 gene (rs355687) were reported to be associated with non-responsiveness to the hepatitis B vaccine^18^. Toll-like receptors (TLRs), which act as bridging molecules between innate and adaptive immunity, are able to recognize different viruses and/or bacteria by pathogen-associated molecular patterns (PAMPs) and activate a pathogen specific immune response^19^,^20^. TLR3, TLR4 and TLR9 are important members of TLR gene family, with TLR3 recognizing virus-derived dsRNA as well as poly I: C, a synthetic dsRNA analogue, TLR4 recognizing lipopolysaccharide, and TLR9 recognizing unmethylated CpG motifs present in bacteria and viruses. Genetic variants in these three genes may influence its biological functions. Rs3775291 in the TLR3 gene was shown to be associated with dsRNA binding capacity^21^, rs1927914 in the TLR4 gene was found to influence the binding sites of transcriptional factor, promoter activity and gene expression^22^, and rs352140 in the TLR9 gene was observed to affect the expression of TLR9 gene^23^. Hence, we hypothesized that the above 10 SNPs in the 9 genes may modify the risk of intrauterine HBV infection.

Thus we performed this study which aimed to investigate the associations between the 10 SNPs and risk of HBV intrauterine infection. Furthermore, we evaluated the associations of these loci from both maternal and neonatal aspects with HBV intrauterine infection, respectively.

## Results

### Characteristics of mothers and neonates

706 mother-neonate pairs were included in this study and 44 neonates were tested positive for HBsAg and/or HBV DNA. The HBV intrauterine infection rate was 6.23% (44/706). Characteristics of mothers and neonates of cases and controls are summarized in Table 1. Mothers in case group were younger than those in control group (*P* = 0.002). While, no statistically significant difference was observed between mothers of cases and controls in the distribution of profession (*P* = 0.804). Additionally, there were no statistically significant differences in birth weight, birth length, gestational weeks and gender between neonates of cases and controls.

### Associations between maternal characteristics and HBV intrauterine transmission

As shown in Table 2, maternal HbeAg positivity (*P* < 0.001), maternal HBV DNA positivity (*P* < 0.001) and caesarean section (*P* = 0.004) were significantly associated with HBV intrauterine transmission. No statistically significant associations were observed between other maternal characteristics and HBV intrauterine transmission.

### Associations between maternal and neonatal SNPs and HBV intrauterine infection

10 SNPs were successfully genotyped, and basic information about these SNPs is shown in Supplementary Table S1. The call rate of all the maternal and neonatal SNPs were >95%, and the genotypes for all SNPs of mothers and neonates in control group conformed to Hard-Weinberg equilibrium (HWE, FDR-*P* > 0.05). The maternal and neonatal minor allele frequencies (MAFs) in the control group were similar to those in the HapMap database of Han Chinese in Beijing except for the SNP (rs3128917) in HLA-DP which were similar to those in HapMap database of Southern Han Chinese. Distribution of genotypes of the 10 SNPs of mothers and neonates was presented in Supplementary Tables S2 and S3. Moreover, rs2296651 and rs7154439 were not in strong LD with each other of mothers (r^2^ = 0.008) or neonates (r^2^ = 0.008) in the control group. As shown in Table 3, after adjusting for maternal age, maternal HBeAg, maternal HBV DNA and mode of delivery, mothers who carried the rs355687 CT genotypes in CXCL13 gene had a lower risk of HBV intrauterine transmission compared to those who carried CC genotypes (OR = 0.25, 95% CI, 0.08–0.82, *P* = 0.022). Besides, a marginally significantly decreased risk of HBV intrauterine transmission was also found under the dominant model (OR = 0.34, 95% CI, 0.11–1.01, *P* = 0.052). Then, we performed the association analysis for each neonatal SNP under general genetic model, dominant, recessive and additive models. Unfortunately, none of the *P* value was statistically significant after adjustment for maternal age, maternal HBeAg, maternal HBV DNA and mode of delivery. However, the rs3130542 in HLA-C gene of neonates was found to be marginally significantly associated with decreased risk of susceptibility to intrauterine HBV infection under the additive model (OR = 0.55, 95% CI, 0.29–1.04, *P* = 0.064).

### Gene-virus interactions with respect to the risk of HBV intrauterine infection

Table 4 shows the results of multiplicative and additive interaction analyses between maternal rs355687, neonatal 3130542 and maternal HBeAg, maternal HBV DNA. Unfortunately, no significant multiplicative or additive interactions were detected.

### Interactions between genes and mode of delivery with respect to the risk of HBV intrauterine infection

The results of multiplicative interaction analyses between maternal rs355687, neonatal 3130542 and mode of delivery are shown in Table 5. No significant multiplicative interactions between genes and mode of delivery were detected.

## Discussion

In this study, we investigated the associations between the genetic variants in 9 candidate genes and risk of HBV intrauterine infection. Our results showed that maternal rs355687 in CXCL13 gene was significantly associated with decreased risk of susceptibility to intrauterine infection of HBV.

Maternal characteristics play an important role in the intrauterine infection of HBV. In our study, we observed that mothers with younger age had a significantly higher risk of HBV intrauterine transmission, which is similar with the results obtained by Schillie *et al*.^24^. The possible explanation is that younger mothers were more often HBeAg positive (Supplementary Table S4) and had longer labors than older mothers^25^. Majority of previous studies investigating the influencing factors of HBV intrauterine infection showed that maternal HBeAg positivity and maternal HBV DNA positivity were two main risk factors^5^,^26^,^27^, which is consistent with our results. Furthermore, we also found that caesarean delivery was a protective factor of HBV intrauterine transmission, which is in agreement with earlier studies^5^,^9^, indicating that caesarean delivery might help to reduce HBV infection rate of neonates intrauterinely.

Apart from these above factors associated with HBV intrauterine infection, recent studies have suggested that host genetic factors may also have an influence on the susceptibility to HBV intrauterine infection^7^,^9^,^28^. In the current study, maternal rs355687 in CXCL13 gene was found to be associated with decreased risk of HBV intrauterine transmission. CXCL13, a molecule of TfH cells, was found to play an integral role in facilitating an effective immune response against HBV by Publicover *et al*.^29^. In addition, their study also showed that CXCL13 expression increases in an age-dependent manner^30^. The rs355687 is located in the intron of CXCL13 gene, variation of which may affect the transcription of CXCL13 gene. Zhaojun Duan *et al*. found that the rs355687 in CXCL13 gene was associated with non-responsiveness to the hepatitis B vaccine, and the CT genotype in rs355687 appeared more frequently in the group defined as HBV responders (*P*  =  0.038, OR = 0.30, 95% CI, 0.09–0.97)^18^. Based on the above findings, we speculated that CT genotype in maternal rs355687 might up-regulate the expression of CXCL13 gene, increase CXCL13 level and clear HBV in the blood of mothers, and thus interrupt HBV transmission from mothers to newborns. Furthermore, no significant association between neonatal rs355687 in CXCL13 gene and HBV intrauterine infection was observed in our study, which was possibly due to the age-dependent manner of CXCL13 production. Notably, we found that the rs3130542 in HLA-C gene of neonates was marginally significantly associated with decreased risk of susceptibility to HBV intrauterine infection under the additive model (OR = 0.55, 95% CI, 0.29–1.04, *P* = 0.064). HLA-C encodes a type I HLA molecule. Increased HLA-C expression in hepatocytes stimulates attack by cytotoxic T cells (CTLs), a crucial component of HBV clearance^31^,^32^. Zhibin Hu *et al*. conducted a genome-wide association studies (GWAS) in Chinese population and identified the A allele of rs3130542 in HLA-C gene was associated with chronic HBV infection (OR = 1.33, *P* = 9.49 × 10^−14^)^15^. And Montgomery SB *et al*. showed that the A allele of rs3130542 was associated with lower expression of HLA-C^33^. Hence, we inferred that neonates carrying the G allele of rs3130542 in HLA-C gene might have a higher expression of HLA-C, which resulted in a decreased risk of HBV intrauterine infection. Subsequently, we ran interaction analyses of the relationship between maternal rs355687, neonatal 3130542 and maternal HBeAg, maternal HBV DNA, mode of delivery and found no significant interactions, which indicated that virological markers (HBeAg, HBV DNA), mode of delivery and host genetic factors might confer susceptibility of HBV intrauterine infection individually. Besides, we did not observe statistically significant associations between other 8 maternal and neonatal SNPs (rs2296651, rs7154439, rs3128917, rs3922, rs3775291, rs1927914, rs352140 and rs4821116) and risk of HBV intrauterine infection. One plausible reason was that the sample size of neonates with HBV intrauterine infection was small in the current study, which resulted in failure to detect the statistically significant associations between them. Another possible explanation was that these SNPs had no effect on the susceptibility to intrauterine infection of HBV essentially.

Some potential limitations in this study should be considered. Firstly, the low incidence of HBV intrauterine infection results in the relatively small sample size of neonates with HBV intrauterine infection, and further studies with larger sample size are needed. Secondly, only one or two SNPs in a gene, reported or identified by previous studies, were selected, so we could not reveal the role of a gene in HBV intrauterine infection comprehensively. Further studies covering all probably functional SNPs in candidate genes are needed. Thirdly, the significant associations found were only at the statistical level and their biological functions were not evaluated. Further biological experiments are therefore warranted to clarify its mechanism underlying the etiology of HBV intrauterine infection, and are among the future studies we plan to pursue.

In summary, our study suggested that maternal rs355687 variant in CXCL13 gene was associated with decreased risk of HBV intrauterine infection. To the best of our knowledge, this is the first study on exploring the roles of host genetic variants in HBV intrauterine infection from both maternal and neonatal aspects. The characterization of functional significance of this particular CXCL13 molecule in HBV intrauterine infection may provide insight leading to the development of novel prophylactic and therapeutic approaches.

## Materials and Methods

### Study subjects

719 HBsAg positive pregnant women were consecutively enrolled in this study from May 2012 to October 2014 at Wuhan Women and Children Medical and Healthcare Center, Wuhan, China. In total, these women delivered 731 neonates, including 12 twins. Women who co-infected with HCV and/or HIV or who gave multiple births were excluded, which yielded 706 mother-neonate pairs eventually.

Maternal information (maternal demographics, characteristics before and during pregnancy), maternal complications, mode of delivery and neonatal characteristics (birth weight, birth length and gender *et al*.) were obtained from medical records. 2-mL procoagulant and 2-mL anticoagulant peripheral venous blood samples of the mothers were collected before delivery, while 2-mL procoagulant and 2-mL anticoagulant femoral venous blood samples of their newborns were collected before the administration of passive-active immunoprophylaxis. Procoagulant blood samples were used for the detection of HBV DNA levels, while anticoagulant blood samples were stored at −80 °C for laboratory testing. After blood collection, the newborns received hepatitis B immune globulin (HBIG) intramuscularly immediately and they were also administered with hepatitis B vaccines at month 0, 1 and 6. This study was approved by the ethics committee of Tongji Medical College of Huazhong University of Science and Technology, and the methods were carried out in accordance with the approved guidelines. Written informed consent was obtained from all mothers.

### Serological HBV markers and HBV DNA

Information about HBV serological markers of mothers which were detected at the prenatal care visit were acquired from their medical records. HBV DNA levels were tested by RT-PCR (Da’an Gene Co. Ltd, Sun Yat-Sen University, Guangdong, China), which was performed by the staffs of clinical laboratory in the hospital. HBsAg of neonates were measured using ELISA (Kehua biotechnology, Shanghai, China). All procedures were performed according to the manufacturers’ instructions. HBV DNA loads >1 × 10^3^ copies/ml were defined as positive. HBV intrauterine infection was defined as HBsAg and/or HBV DNA positive in neonatal peripheral venous blood within 24 hours of birth and before administration of active or passive immune prophylaxis^34^. Newborns with intrauterine HBV infection were classified as case group, those without were classified as control group.

### DNA extraction and genotyping

Genomic DNA was extracted from the blood samples using a TIANamp Blood DNA Kit DP318 (TIANGEN BIOTECH (BEIJING) CO., LTD, China) under the instruction of manufacturer. DNA concentration and optical density were determined using a NanoDrop 1000 spectrophotometer (Thermo Fisher Scientific, Waltham, Massachusetts, USA). SNPs of each sample were genotyped by the TaqMan SNP Genotyping Assay (Applied Biosystems, Foster City, CA, USA) with a 7900 HT Fast Real-Time PCR System (Applied Biosystems, Foster City, CA, USA) according to the manufacturer’s instructions. For quality control, genotyping was performed without knowing case control status. In addition, samples with a call rate <80% were removed to make sure the call rate of each SNP was over 95%.

### Statistical analysis

The Hardy-Weinberg equilibrium (HWE) for genotypes was assessed by a goodness-of-fit *χ*^2^ test among controls. The difference in distribution of demographic characteristics, genotype frequencies between cases and controls were examined by Pearson’s *χ*^2^ test or t-test, when appropriate. The association between each SNP of mothers and neonates and risk of intrauterine HBV infection was evaluated by the odds ratio (OR) and its 95% confidence interval (CI) using unconditional multivariable logistic regression model with adjustment for maternal age, maternal HBeAg, maternal HBV DNA level and mode of delivery. In order to avoid the assumption of genetic models, general genetic model, dominant, recessive and additive models were all calculated. Multiplicative and additive interactions were tested to evaluate interactions between genes and virological markers (HBeAg and HBV DNA). Multiplicative interaction was tested to evaluate interactions between genes and mode of delivery. Multiplicative interactions were evaluated by a likelihood ratio test in the logistic regression model. Synergy index (SI), attributable proportion due to interaction (AP), and relative excess risk due to interaction (RERI) and its 95% CIs were calculated in additive interaction analyses. Lack of interaction is indicated by 95% CI of SI containing 1 and 95% CIs of AP and RERI containing 0. All statistical analyses were conducted by SPSS v18.0 (SPSS, Chicago, Illinois, USA). Linkage disequilibrium (LD) was performed using the Haploview v4.2 software, by determining D’ and γ^2^ values. The additive interaction analysis was performed using the excel sheet made by Andersson *et al*.^35^. All *P* values were two-tailed with a statistically significant level set at 0.05. False discovery test was performed to take into account the multiple comparisons.

## Additional Information

**How to cite this article**: Wan, Z. *et al*. Genetic variant in CXCL13 gene is associated with susceptibility to intrauterine infection of hepatitis B virus. *Sci. Rep.* **6**, 26465; doi: 10.1038/srep26465 (2016).

## Supplementary Material

Supplementary Information

## Figures and Tables

**Table 1:** Characteristics of mothers and neonates enrolled in the study.

	**Case neonates (n = 44)**	**Control neonates (n = 662)**	***P***
Maternal Characteristics			
Age (years)	26.43 ± 4.00	28.36 ± 3.99	**0.002**
Profession			0.804
Employed	20/42 (47.6)	299/655 (45.6)	
Unemployed	22/42 (52.4)	356/655 (54.4)	
Infant characteristics			
Birth weight (g)	3245 ± 434	3296 ± 401	0.418
Birth length (cm)	50.21 ± 1.49	50.14 ± 1.56	0.771
Gestational weeks (w)	39.39 ± 1.10	39.04 ± 1.34	0.101
Gender			0.073
Male	18/44 (40.9)	358/653 (54.8)	
Female	26/44 (59.1)	295/653 (45.2)	

**Table 2:** Univariate analyses of associations between maternal characteristics and HBV intrauterine transmission.

**Characteristics**	**Case neonates (n = 44)**	**Control neonates (n = 662)**	***P***
Pre-pregnancy			
Nulliparas	39/43 (90.7)	516/657 (78.5)	0.057
Menstrual regularity	41/41 (100.0)	591/608 (97.2)	0.562
History of abortion	13/44 (29.5)	285/662 (43.1)	0.079
Family history of HBV infection	2/44 (4.5)	37/662 (5.6)	1.000
Pregnancy			
Maternal HBeAg positive	27/44 (61.4)	129/628 (20.5)	**<0.001**
Maternal HBV DNA positive	23/35 (65.7)	185/526 (35.2)	**<0.001**
Number of prenatal care visits			0.429
<10	17/37 (45.9)	232/589 (39.4)	
≥10	20/37 (54.1)	357/589 (60.6)	
Influenza and/or fever in the first trimester	7/44 (15.9)	111/662 (16.8)	0.883
Colporrhagia in the first trimester	6/44 (13.6)	129/662 (19.5)	0.339
Pregnancy-induced hypertension	3/44 (6.8)	15/662 (2.3)	0.173
Delivery			
Cesarean section	21/44 (47.7)	454/660 (68.8)	**0.004**
Preterm birth (<37 weeks)	2/43 (4.7)	28/657 (4.3)	1.000
Premature rupture of membranes	4/44 (9.1)	111/662 (16.8)	0.182
Fetal distress	5/44 (11.4)	75/662 (11.3)	1.000
Abnormality of umbilical cord	10/44 (22.7)	172/662 (26.0)	0.633
Praevia placenta	0/44 (0)	22/662 (3.3)	0.435

**Table 3:** Association between maternal and neonatal candidate SNPs and HBV intrauterine infection.

**Genotypes**	**Cases**	**Controls**	OR (95% CI)*	***P***
Mothers				
rs355687				
CC	5 (14.3)	59 (11.1)	1	
CT	12 (34.3)	253 (47.7)	0.25 (0.08–0.82)	**0.022**
TT	18 (51.4)	218 (41.1)	0.45 (0.14–1.41)	0.172
Dominant			0.34 (0.11–1.01)	**0.052**
Recessive			1.39 (0.67–2.90)	0.376
Additive			0.96 (0.53–1.76)	0.904
Neonates				
rs3130542				
AA	2 (4.8)	25 (3.8)	1	
AG	16 (38.1)	218 (33.3)	0.51 (0.10–2.73)	0.434
GG	24 (57.1)	412 (62.9)	0.29 (0.06–1.51)	0.140
Dominant			0.36 (0.07–1.82)	0.219
Recessive			0.52 (0.24–1.13)	0.097
Additive			0.55 (0.29–1.04)	**0.064**

^*^Adjusted by maternal age, maternal HBeAg, maternal HBV DNA and mode of delivery in the unconditional logistic regression.

**Table 4:** Analysis of interactions between HBeAg status, HBV DNA status and maternal *rs355687*, neonatal *rs3130542* and risk of HBV intrauterine transmission.

	**Genotype**	**Cases**	**Controls**	OR (95% CI)*	***P***_**mul**_	**SI (95% CI)**	**AP (95% CI)**	**RERI (95% CI)**
Mothers								
HBeAg								
Negative	CT+TT	11 (31.4)	358 (70.9)	1	0.758	1.63 (0.13–20.81)	0.36 (−1.15–1.86)	4.48 (−24.60–33.55)
Positive	CT+TT	19 (54.3)	90 (17.8)	5.86 (2.33–14.75)				
Negative	CC	4 (11.4)	52 (10.3)	3.23 (0.95–10.99)				
Positive	CC	1 (2.9)	5 (1.0)	12.57 (1.15–137.92)				
HBV DNA								
Negative	CT+TT	8 (22.9)	294 (57.0)	1	0.206	0.19 (0.01–5.29)	−2.91 (−12.37–6.55)	−8.92 (−28.35–10.50)
Positive	CT+TT	22 (62.9)	166 (32.2)	2.47 (0.93–6.60)				
Negative	CC	4 (11.4)	41 (7.9)	5.12 (1.40–18.70)				
Positive	CC	1 (2.9)	15 (2.9)	2.51 (0.27–23.67)				
Neonates								
HBeAg								
Negative	GG	7 (16.7)	311 (50.1)	1	0.425	1.14 (0.35–3.72)	0.11 (−0.82–1.04)	1.03 (−8.34–10.40)
Positive	GG	17 (40.5)	85 (13.7)	6.57 (2.09–20.65)				
Negative	AA+AG	9 (21.4)	184 (29.6)	2.62 (0.88–7.80)				
Positive	AA+AG	9 (21.4)	41 (6.6)	9.22 (2.53–33.62)				
HBV DNA								
Negative	GG	7 (16.7)	262 (40.7)	1	0.702	2.06 (0.26–16.20)	0.37 (−0.36–1.10)	1.30 (−1.97–4.56)
Positive	GG	17 (40.5)	143 (22.2)	1.80 (0.60–5.36)				
Negative	AA+AG	6 (14.3)	163 (25.3)	1.59 (0.51–4.88)				
Positive	AA+AG	12 (28.6)	76 (11.8)	2.98 (0.95–9.41)				

*P*_mul_ was calculated using the multiplicative interaction term.

^*^Adjusted by maternal age, mode of delivery and maternal HBV DNA or maternal HBeAg.

**Table 5:** Analysis of interactions between mode of delivery and maternal *rs355687*, neonatal *rs3130542* and risk of HBV intrauterine transmission.

	**Genotype**	**Cases**	**Controls**	OR (95% CI)*	***P***_**mul**_
Mothers					
Mode of delivery					
Cesarean section	CT+TT	11 (31.4)	325 (61.4)	1	0.278
Vaginal delivery	CT+TT	19 (54.3)	145 (27.4)	4.82 (2.09–11.12)	
Cesarean section	CC	4 (11.4)	47 (8.9)	4.63 (1.28–16.69)	
Vaginal delivery	CC	1 (2.9)	12 (2.3)	5.66 (0.60–53.25)	
Neonates					
Mode of delivery					
Cesarean section	GG	11 (26.2)	276 (42.3)	1	0.972
Vaginal delivery	GG	13 (31.0)	135 (20.7)	3.52 (1.26–9.87)	
Cesarean section	AA+AG	10 (23.8)	176 (27.0)	1.93 (0.66–5.64)	
Vaginal delivery	AA+AG	8 (19.0)	66 (10.1)	6.63 (2.04–21.56)	

*P*_mul_ was calculated using the multiplicative interaction term.

^*^Adjusted by maternal age, maternal HBeAg and maternal HBV DNA.

## References

[b1] Dienstag J. L. Hepatitis B virus infection. N Engl J Med 359, 1486–1500 (2008). 18832247 10.1056/NEJMra0801644

[b2] Liang X. . Epidemiological serosurvey of hepatitis B in China–declining HBV prevalence due to hepatitis B vaccination. Vaccine 27, 6550–6557 (2009). 19729084 10.1016/j.vaccine.2009.08.048

[b3] Zhang S. L., Yue Y. F., Bai G. Q., Shi L. & Jiang H. Mechanism of intrauterine infection of hepatitis B virus. World J Gastroenterol 10, 437–438 (2004). 14760774 10.3748/wjg.v10.i3.437 PMC4724928

[b4] Shao Z. J. . Mother-to-infant transmission of hepatitis B virus: a Chinese experience. J Med Virol 83, 791–795 (2011). 21360547 10.1002/jmv.22043

[b5] Guo Z. . Risk factors of HBV intrauterine transmission among HBsAg-positive pregnant women. J Viral Hepat 20, 317–321 (2013). 23565613 10.1111/jvh.12032 PMC3623007

[b6] Wen W. H. . Mother-to-infant transmission of hepatitis B virus infection: significance of maternal viral load and strategies for intervention. J Hepatol 59, 24–30 (2013). 23485519 10.1016/j.jhep.2013.02.015

[b7] Yu H., Zhu Q. R., Gu S. Q. & Fei L. E. Relationship between IFN-gamma gene polymorphism and susceptibility to intrauterine HBV infection. World J Gastroenterol 12, 2928–2931 (2006). 16718821 10.3748/wjg.v12.i18.2928 PMC4087813

[b8] Xu Y. Y. . Association between the frequency of class II HLA antigens and the susceptibility to intrauterine infection of hepatitis B virus. Int J Biol Sci 4, 111–115 (2008). 18463715 10.7150/ijbs.4.111 PMC2359901

[b9] Gao Y. . Evaluation of neonatal Toll-like receptors 3 (c.1377C/T) and 9 (G2848A) gene polymorphisms in HBV intrauterine transmission susceptibility. Epidemiol Infect 143, 1868–1875 (2015). 25388852 10.1017/S0950268814002921 PMC9507260

[b10] Yan H. . Sodium taurocholate cotransporting polypeptide is a functional receptor for human hepatitis B and D virus. Elife 1, e49 (2012). 10.7554/eLife.00049 PMC3485615 23150796

[b11] Peng L. . The p.Ser267Phe variant in SLC10A1 is associated with resistance to chronic hepatitis B. Hepatology 61, 1251–1260 (2015). 25418280 10.1002/hep.27608

[b12] Su Z. . Association of the gene polymorphisms in sodium taurocholate cotransporting polypeptide with the outcomes of hepatitis B infection in Chinese Han population. Infect Genet Evol 27, 77–82 (2014). 25010264 10.1016/j.meegid.2014.07.001

[b13] Kamatani Y. . A genome-wide association study identifies variants in the HLA-DP locus associated with chronic hepatitis B in Asians. Nat Genet 41, 591–595 (2009). 19349983 10.1038/ng.348

[b14] Wong D. K. . Role of HLA-DP polymorphisms on chronicity and disease activity of hepatitis B infection in Southern Chinese. Plos One 8, e66920 (2013). 23825586 10.1371/journal.pone.0066920 PMC3692552

[b15] Hu Z. . New loci associated with chronic hepatitis B virus infection in Han Chinese. Nat Genet 45, 1499–1503 (2013). 24162738 10.1038/ng.2809

[b16] Crotty S. Follicular helper CD4 T cells (TFH). Annu Rev Immunol 29, 621–663 (2011). 21314428 10.1146/annurev-immunol-031210-101400

[b17] Xing T., Xu H. & Yu W. Role of T follicular helper cells and their associated molecules in the pathogenesis of chronic hepatitis B virus infection. Exp Ther Med 5, 885–889 (2013). 23407366 10.3892/etm.2012.864 PMC3570220

[b18] Duan Z. . Genetic polymorphisms of CXCR5 and CXCL13 are associated with non-responsiveness to the hepatitis B vaccine. Vaccine 32, 5316–5322 (2014). 25077417 10.1016/j.vaccine.2014.07.064

[b19] Boehme K. W. & Compton T. Innate sensing of viruses by toll-like receptors. J Virol 78, 7867–7873 (2004). 15254159 10.1128/JVI.78.15.7867-7873.2004 PMC446107

[b20] Takeda K. & Akira S. Toll-like receptors in innate immunity. Int Immunol 17, 1–14 (2005). 15585605 10.1093/intimm/dxh186

[b21] Zhou P., Fan L., Yu K. D., Zhao M. W. & Li X. X. Toll-like receptor 3 C1234T may protect against geographic atrophy through decreased dsRNA binding capacity. Faseb J 25, 3489–3495 (2011). 21712495 10.1096/fj.11-189258

[b22] Hsieh Y. Y., Wan L., Chang C. C., Tsai C. H. & Tsai F. J. STAT2*C related genotypes and allele but not TLR4 and CD40 gene polymorphisms are associated with higher susceptibility for asthma. Int J Biol Sci 5, 74–81 (2009). 19159017 10.7150/ijbs.5.74 PMC2615545

[b23] Xu C. J. . Association study of a single nucleotide polymorphism in the exon 2 region of toll-like receptor 9 (TLR9) gene with susceptibility to systemic lupus erythematosus among Chinese. Mol Biol Rep 36, 2245–2248 (2009). 19130296 10.1007/s11033-008-9440-z

[b24] Schillie S. . Outcomes of infants born to women infected with hepatitis B. Pediatrics 135, e1141–e1147 (2015). 25896839 10.1542/peds.2014-3213

[b25] Marion S. A., Tomm P. M., Pi D. W. & Mathias R. G. Long-term follow-up of hepatitis B vaccine in infants of carrier mothers. Am J Epidemiol 140, 734–746 (1994). 7942775 10.1093/oxfordjournals.aje.a117321

[b26] Lv N. . Analysis on the outcomes of hepatitis B virus perinatal vertical transmission: nested case-control study. Eur J Gastroenterol Hepatol 26, 1286–1291 (2014). 25171026 10.1097/MEG.0000000000000189

[b27] Bai H. . Relationship of hepatitis B virus infection of placental barrier and hepatitis B virus intra-uterine transmission mechanism. World J Gastroenterol 13, 3625–3630 (2007). 17659715 10.3748/wjg.v13.i26.3625 PMC4146804

[b28] Xu Y. Y. . Association between the frequency of class II HLA antigens and the susceptibility to intrauterine infection of hepatitis B virus. Int J Biol Sci 4, 111–115 (2008). 18463715 10.7150/ijbs.4.111 PMC2359901

[b29] Publicover J. . Age-dependent hepatic lymphoid organization directs successful immunity to hepatitis B. J Clin Invest 123, 3728–3739 (2013). 23925290 10.1172/JCI68182 PMC3754256

[b30] Leavy O. Antiviral immunity: A ‘mature’ way of controlling HBV. Nat Rev Immunol 13, 616 (2013). 23954937 10.1038/nri3527

[b31] Ballardini G. . HLA-A,B,C, HLA-D/DR and HLA-D/DQ expression on unfixed liver biopsy sections from patients with chronic liver disease. Clin Exp Immunol 70, 35–46 (1987). 3319302 PMC1542210

[b32] Zhang Y. . Hepatitis B virus core antigen epitopes presented by HLA-A2 single-chain trimers induce functional epitope-specific CD8+ T-cell responses in HLA-A2.1/Kb transgenic mice. Immunology 121, 105–112 (2007). 17244158 10.1111/j.1365-2567.2007.02543.x PMC2265916

[b33] Montgomery S. B. . Transcriptome genetics using second generation sequencing in a Caucasian population. Nature 464, 773–777 (2010). 20220756 10.1038/nature08903 PMC3836232

[b34] Li X. M. . Effect of hepatitis B immunoglobulin on interruption of HBV intrauterine infection. World J Gastroenterol 10, 3215–3217 (2004). 15457579 10.3748/wjg.v10.i21.3215 PMC4611277

[b35] Andersson T., Alfredsson L., Kallberg H., Zdravkovic S. & Ahlbom A. Calculating measures of biological interaction. Eur J Epidemiol 20, 575–579 (2005). 16119429 10.1007/s10654-005-7835-x

