# Supplementary material for: Genetic variant in CXCL13 gene is associated with susceptibility to intrauterine infection of hepatitis B virus
Source: Sci Rep. 2016 May 23;6:26465. doi: 10.1038/srep26465 (PMC4876436; doi:10.1038/srep26465)
Supplement: Supplementary Information [file srep26465-s1.pdf]

Genetic variant in CXCL13 gene is associated with susceptibility to intrauterine infection of hepatitis B virus

Zhihua Wan, Xiaofang Lin, Tongyang Li, Aifen Zhou, Mei Yang, Dan Hu, Li Feng, Songxu Peng, Linlin Fan, Si Tu, Bin Zhang, Yukai Du

Supplementary Table S1. Basic information of the SNPs in the current study

Gene	SNP	Position	Alleles	Minor allele	MAF in CHB	MAF in control*	Call rate*	HWE*	FDR-HWE*	MAF in control†	Call rate †	HWE†	FDR-HWE†
SLC10A1	rs2296651	14:69314946	A/G	A	0.029	0.043	0.995	0.307	0.768	0.040	0.997	0.290	0.967
SLC10A1	rs7154439	14:69335581	A/G	A	0.180	0.157	0.995	0.320	0.640	0.159	0.994	0.182	1.000
HLA-DP	rs3128917	6:33167974	G/T	G	0.461	0.606	0.981	0.863	0.959	0.566	0.980	0.992	0.992
HLA-C	rs3130542	6:31340090	A/G	A	0.180	0.200	0.998	0.297	0.990	0.205	0.994	0.562	0.937
CXCR5	rs3922	11:118270810	A/G	G	0.350	0.303	0.989	0.950	0.950	0.308	0.990	0.411	1.000
CXCL13	rs355687	4:78724602	C/T	C	0.340	0.350	0.996	0.258	1.000	0.345	0.993	0.645	0.921
TLR3	rs3775291	4:187241068	C/T	T	0.291	0.312	0.993	0.388	0.554	0.317	0.993	0.800	1.000
TLR4	rs1927914	9:119504546	A/G	G	0.418	0.385	1.000	0.360	0.600	0.390	0.990	0.258	1.000
TLR9	rs352140	3:52231737	C/T	T	0.422	0.344	0.989	0.136	1.000	0.368	0.994	0.894	0.993
UBE2L3	rs4821116	22:20303319	C/T	T	0.359	0.394	1.000	0.525	0.656	0.406	0.997	0.416	0.832

Abbreviations: MAF, minor allele frequency; CHB, Han Chinese in Beijing, China; HWE: Hardy-Weinberg equilibrium; FDR, false discovery rate.

*Calculated among mothers.

†Calculated among neonates.

Supplementary Table S2. Association between maternal candidate SNPs and HBV intrauterine infection

Genotypes	Cases	Controls	OR (95% CI)*	*P*	FDR-*P*†
rs2296651					
GG	32 (91.4)	484 (91.5)	1		
AG	3 (8.6)	45 (8.5)	1.03 (0.29-3.69)	0.964	
rs7154439					0.845
GG	24 (68.6)	373 (70.5)	1		
AG	11 (31.4)	146 (27.6)	0.97 (0.44-2.15)	0.939	
AA	0 (0)	10 (1.9)	0	0.999	
Dominant			0.92 (0.42-2.04)	0.845	
Recessive			0	0.999	
Additive			0.88 (0.41-1.86)	0.734	
rs3128917					1.000
GG	15 (42.9)	192 (36.9)	1		
GT	11 (31.4)	247 (47.4)	0.68 (0.29-1.58)	0.365	
TT	9 (25.7)	82 (15.7)	1.49 (0.58-3.82)	0.409	
Dominant			0.89 (0.43-1.88)	0.766	
Recessive			1.80 (0.76-4.26)	0.185	
Additive			1.14 (0.69-1.88)	0.617	
rs3130542					0.937
AA	1 (2.9)	25 (4.7)	1		
AG	12 (34.3)	162 (30.5)	1.52 (0.17-13.31)	0.704	
GG	22 (62.9)	344 (64.8)	1.14 (0.14-9.55)	0.904	
Dominant			1.26 (0.15-10.28)	0.833	

Recessive			0.78 (0.36-1.67)	0.523	
Additive			0.86 (0.45-1.63)	0.638	
rs3922					1.000
GG	2 (6.3)	49 (9.3)	1		
AG	17 (53.1)	223 (42.2)	2.56 (0.52-12.51)	0.247	
AA	13 (40.6)	257 (48.6)	1.19 (0.24-5.93)	0.830	
Dominant			1.72 (0.37-7.96)	0.490	
Recessive			0.54 (0.25-1.19)	0.129	
Additive			0.79 (0.45-1.38)	0.398	
rs355687					0.468
CC	5 (14.3)	59 (11.1)	1		
CT	12 (34.3)	253 (47.7)	0.25 (0.08-0.82)	**0.022**	
TT	18 (51.4)	218 (41.1)	0.45 (0.14-1.41)	0.172	
Dominant			0.34 (0.11-1.01)	**0.052**	
Recessive			1.39 (0.67-2.90)	0.376	
Additive			0.96 (0.53-1.76)	0.904	
rs3775291					1.000
CC	18 (51.4)	246 (46.6)	1		
TC	17 (48.6)	235 (44.5)	0.88 (0.42-1.85)	0.736	
TT	0 (0)	47 (8.9)	0	0.997	
Dominant			0.74 (0.35-1.54)	0.413	
Recessive			0	0.997	
Additive			0.63 (0.34-1.19)	0.153	
rs1927914					1.000
GG	5 (14.3)	84 (15.8)	1		

AG	17 (48.6)	242 (45.5)	0.90 (0.31-2.64)	0.846	
AA	13 (37.1)	206 (38.7)	0.89 (0.29-2.72)	0.836	
Dominant			0.89 (0.32-2.48)	0.830	
Recessive			0.97 (0.45-2.06)	0.926	
Additive			0.95 (0.56-1.63)	0.860	
rs352140					0.922
CC	13 (37.1)	234 (44.5)	1		
CT	18 (51.4)	222 (42.2)	1.32 (0.60-2.91)	0.488	
TT	4 (11.4)	70 (13.3)	1.16 (0.35-3.84)	0.809	
Dominant			1.29 (0.61-2.72)	0.512	
Recessive			1.00 (0.33-3.08)	0.995	
Additive			1.14 (0.67-1.94)	0.630	
rs4821116					1.000
TT	3 (8.6)	86 (16.2)	1		
CT	15 (42.9)	247 (46.4)	1.78 (0.48-6.60)	0.387	
CC	17 (48.6)	199 (37.4)	2.01 (0.54-7.49)	0.297	
Dominant			1.89 (0.54-6.61)	0.320	
Recessive			1.28 (0.60-2.69)	0.524	
Additive			1.31 (0.76-2.27)	0.336	

*Adjusted by maternal age, maternal HBeAg, maternal HBV DNA and mode of delivery in the unconditional logistic regression.

†*P* value for logistic regression in dominant model modified by FDR.

Supplementary Table S3. Association between neonatal candidate SNPs and HBV intrauterine transmission

Genotypes	Cases	Controls	OR (95% CI)*	*P*	FDR-*P*†
rs2296651					
GG	41 (93.2)	603 (92.1)	1		
GA	3 (6.8)	52 (7.9)	1.25 (0.34-4.67)	0.738	
rs7154439					1.000
GG	30 (68.2)	457 (70.0)	1		
GA	13 (29.5)	184 (28.2)	0.91 (0.41-2.01)	0.812	
AA	1 (2.3)	12 (1.8)	1.00 (0.10-9.59)	0.998	
Dominant			0.92 (0.42-1.98)	0.821	
Recessive			1.03 (0.11-9.74)	0.979	
Additive			0.93 (0.47-1.86)	0.847	
rs3128917					0.594
GG	16 (36.4)	206 (32.0)	1		
TG	16 (36.4)	316 (49.1)	0.85 (0.35-2.05)	0.720	
TT	12 (27.3)	121 (18.8)	1.80 (0.70-4.61)	0.221	
Dominant			1.13 (0.51-2.48)	0.766	
Recessive			1.97 (0.88-4.42)	0.100	
Additive			1.33 (0.81-2.20)	0.264	
rs3130542					0.576
AA	2 (4.8)	25 (3.8)	1		
AG	16 (38.1)	218 (33.3)	0.51 (0.10-2.73)	0.434	
GG	24 (57.1)	412 (62.9)	0.29 (0.06-1.51)	0.140	
Dominant			0.36 (0.07-1.82)	0.219	

Recessive			0.52 (0.24-1.13)	0.097	
Additive			0.55 (0.29-1.04)	0.064	
rs3922					0.770
GG	4 (9.1)	66 (10.2)	1		
AG	16 (36.4)	268 (41.2)	1.01 (0.27-3.86)	0.984	
AA	24 (54.5)	316 (48.6)	0.77 (0.21-2.89)	0.700	
Dominant			0.87 (0.24-3.10)	0.831	
Recessive			0.76 (0.37-1.59)	0.469	
Additive			0.83 (0.48-1.45)	0.513	
rs355687					0.801
CC	7 (15.9)	81 (12.4)	1		
TC	16 (36.4)	290 (44.5)	1.36 (0.39-4.71)	0.628	
TT	21 (47.7)	281 (43.1)	1.58 (0.48-5.25)	0.452	
Dominant			1.48 (0.47-4.71)	0.503	
Recessive			1.26 (0.61-2.62)	0.535	
Additive			1.23 (0.72-2.09)	0.445	
rs3775291					0.378
CC	26 (59.1)	303 (46.5)	1		
TC	15 (34.1)	285 (43.7)	0.53 (0.24-1.15)	0.107	
TT	3 (6.8)	64 (9.8)	0.54 (0.12-2.51)	0.430	
Dominant			0.53 (0.25-1.11)	0.092	
Recessive			0.71 (0.16-3.21)	0.656	
Additive			0.61 (0.33-1.15)	0.126	
rs1927914					0.986
GG	11 (25.0)	92 (14.2)	1		

GA	15 (34.1)	323 (49.7)	0.51 (0.19-1.37)	0.179	
AA	18 (40.9)	235 (36.2)	0.90 (0.33-2.42)	0.831	
Dominant			0.66 (0.27-1.61)	0.359	
Recessive			1.44 (0.68-3.06)	0.338	
Additive			1.04 (0.61-1.77)	0.876	
rs352140					0.885
CC	19 (43.2)	262 (40.1)	1		
CT	16 (36.4)	302 (46.2)	0.55 (0.24-1.27)	0.160	
TT	9 (20.5)	89 (13.6)	1.40 (0.53-3.68)	0.493	
Dominant			0.74 (0.35-1.54)	0.415	
Recessive			1.88 (0.77-4.60)	0.168	
Additive			1.04 (0.62-1.75)	0.885	
rs4821116					0.428
TT	4 (9.1)	103 (15.7)	1		
CT	19 (43.2)	326 (49.8)	2.10 (0.45-9.82)	0.347	
CC	21 (47.7)	226 (34.5)	3.18 (0.68-14.93)	0.142	
Dominant			2.54 (0.57-11.37)	0.222	
Recessive			1.70 (0.82-3.54)	0.154	
Additive			1.65 (0.92-2.96)	0.095	

[*]Adjusted by maternal age, maternal HBeAg, maternal HBV DNA and mode of delivery in the unconditional logistic regression.

[†]*P* value for logistic regression in additive model modified by FDR.

Supplementary Table S4. Association between maternal age and maternal HBeAg status

	Maternal age (years)		*P*
	<25	≥25	
Maternal HBeAg			<0.001
Positive	42 (42.0)	113 (20.0)	
Negative	58 (58.0)	453 (80.0)	
